# Fluorescent labeling of asbestos fiber for enhanced asbestos detection under fluorescence microscopy

**DOI:** 10.3389/fpubh.2025.1568581

**Published:** 2025-05-21

**Authors:** Akio Kuroda

**Affiliations:** Graduate School of Integrated Sciences for Life, Hiroshima University, Hiroshima, Japan

**Keywords:** asbestos, fluorescence, fluorescent labeling, fluorescence microscopy, asbestos-binding protein

## Abstract

The application of fluorescence microscopy (FM) for detecting micro- and nano-scale inorganic materials has historically been limited by the lack of specific fluorescent probes. However, recent research has demonstrated that asbestos-binding proteins can act as effective fluorescent probes, significantly enhancing the sensitivity and selectivity of FM for asbestos fiber detection. This advancement enables the identification of nano-scale fibers at lower magnifications, reducing the labor costs associated with asbestos contamination detection. Based on these advantages, two FM-based methods have been developed: (i) phase-contrast microscopy (PCM)-FM, a differential counting approach fully compatible with PCM-based epidemiological data, and (ii) portable FM, which shows strong potential for rapid on-site asbestos screening. Additionally, FM may enable multicolor labeling and live-cell fluorescent imaging of asbestos, opening new avenues for asbestos research. Despite these advancements, several challenges remain. Fluorescent probes alone cannot definitively identify asbestos, and issues such as cross-reactivity need to be addressed. This review highlights future perspectives and challenges for advancing FM methods in asbestos detection and research.

## 1 Introduction

**Figure 1:**
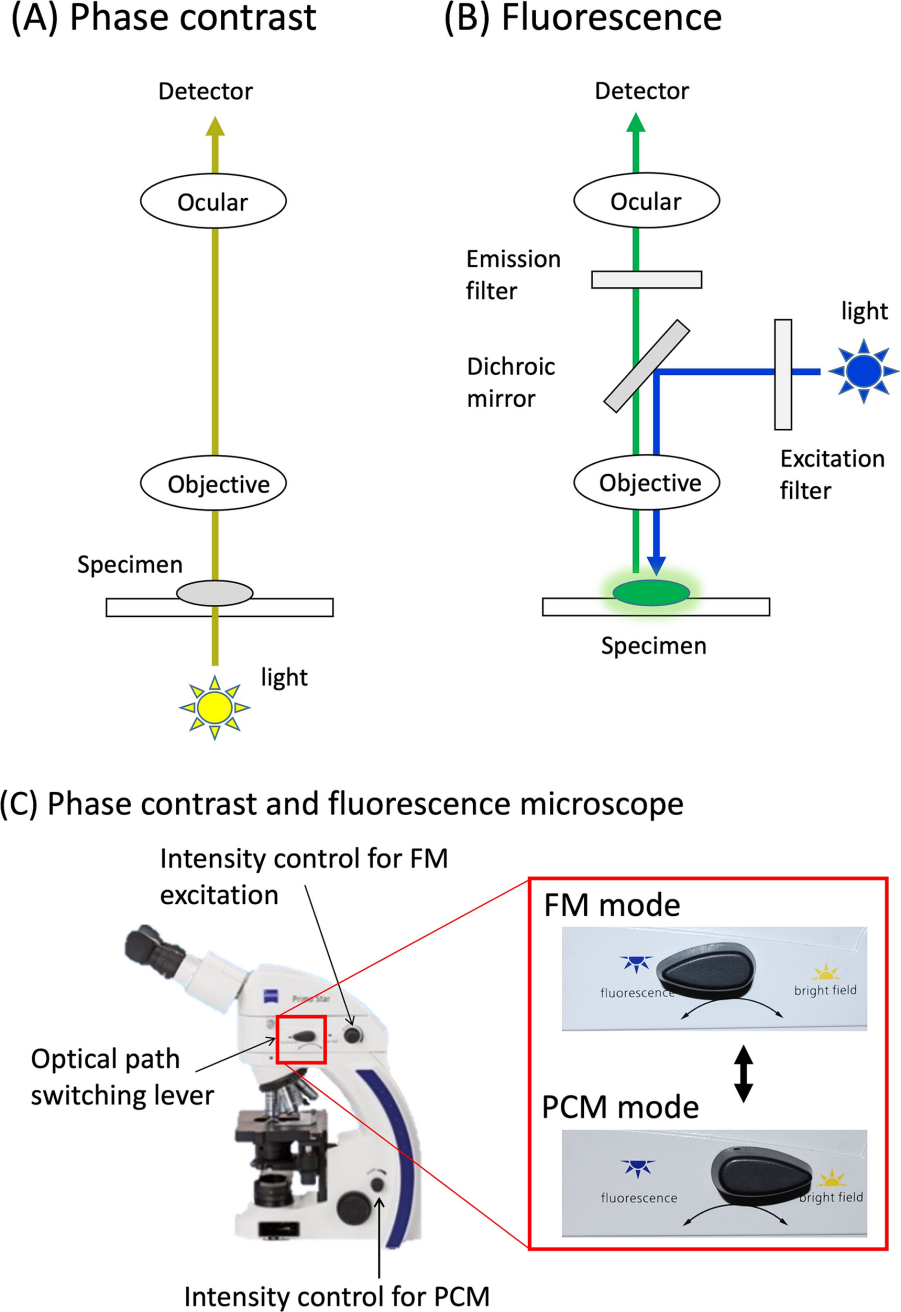
Phase contrast and fluorescence microscope. Optical pathways of phase contrast **(A)** and fluorescence mode **(B)** are illustrated. These pathways are easily switched by a switch lever on the microscope **(C)**.

Fluorescence microscopy (FM) is an essential analytical tool in modern life sciences (1, 2). In contrast to phase-contrast microscopy (PCM), the excitation light in FM illuminates the specimen from above and passes through the objective lens (Figures 1A,B). The fluorescence emitted by the specimen, which has a longer wavelength than the excitation light, is then focused on the detector through the same objective. A dichroic mirror serves as a wavelength-specific filter, transmitting the fluorescent light to the detector while reflecting any remaining excitation light back toward the source. The emission filter also transmits the fluorescent light while blocking the excitation light, ensuring a dark background and resulting in a high signal-to-noise (S/N) ratio. This set-up offers several critical advantages: (i) High sensitivity: FM can detect extremely low quantities of molecules, including single molecules, owing to its high S/N ratio; (ii) Specificity: Immunofluorescence techniques with fluorescently labeled antibodies enable precise visualization of the distribution, abundance, and interactions of targets within complex environments; (iii) Dynamic studies: Live-cell imaging enables real-time observation of molecular interactions and cellular processes, such as protein trafficking, cell signaling, and mitosis, without disrupting the sample; and (iv) Multicolor imaging: The use of multiple fluorescent dyes simultaneously facilitates the visualization of several targets in the same sample, revealing complex molecular networks.

Although these advantages make FM indispensable for advancing biological research, its application to micro- and nano-scale inorganic materials is limited by the scarcity of specific fluorescent probes. Developing such probes could unlock the potential of FM for detecting micro- and nano-scale inorganic materials, including asbestos fibers. For example, the widely used PCM method for airborne asbestos detection has notable limitations such as its inability to distinguish asbestos fibers from non-asbestos fibers and its low sensitivity for detecting thin chrysotile fibers (3, 4). Transmission electron microscopy (TEM) and scanning electron microscopy (SEM) are considered gold standards for identifying asbestos fibers because they provide high-resolution morphological imaging and elemental composition analysis. Additionally, TEM offers crystallographic analysis, enabling precise identification of asbestos types. However, the widespread use of electron microscopy significantly increases the cost and time required for asbestos analysis, making comprehensive asbestos monitoring impractical in many occupational settings. FM has the potential to overcome these challenges. However, effective detection of asbestos fibers using FM requires probes that (i) possess sufficient binding affinity to visualize extremely thin asbestos fibers and (ii) exhibit high specificity for distinguishing asbestos fibers from non-asbestos fibers, particularly those commonly used in the construction industry.

Material-binding peptides were first identified by Brown et al. using the cell surface display method (5, 6). Subsequently, random peptide libraries created using the more convenient phage display method were utilized to select binding peptides for various inorganic materials. Whaley et al. visualized ultrathin gallium arsenide (GaAs) lines on a GaAs/SiO_2_-patterned substrate using FM and a phage displaying a GaAs-binding peptide (7). Notably, this peptide selectively binds to the [100] GaAs face, but not to the [111] face, highlighting the potential for peptides or proteins to selectively adhere to specific inorganic surfaces. This preferential attachment of fluorescently labeled material-binding peptides to surfaces with defined chemical and structural compositions allows for the selective visualization of target materials under FM.

This review details the exploration of proteins and peptides that bind to asbestos, emphasizing their sensitivity and specificity when labeled with fluorescent dyes. Efforts to leverage these fluorescently labeled proteins and peptides for developing PCM-FM differential counting and portable FM methods are also outlined. Furthermore, the application of multicolor labeling, live-cell fluorescent imaging of asbestos, and the future perspectives and challenges associated with FM methods are discussed.

## 2 Asbestos-binding proteins

Asbestos is generally categorized into two mineral groups: serpentine and amphibole, which differ in crystal structure and toxicity (4, 8–10). Chrysotile, the only asbestos type in the serpentine group, is characterized by curly and flexible fibers with a positively charged surface under physiological conditions. Chrysotile accounts for over 90% of the asbestos used industrially, making it the most common type encountered in various applications (4). In contrast, the five types of amphibole fibers (actinolite, amosite, anthophyllite, crocidolite, and tremolite) have rod- or needle-like fibers that are more brittle than chrysotile. Their surfaces are primarily composed of crystalline silica, which consists of double chains of silica tetrahedra and carries a negative charge. These structural differences contribute to the higher toxicity of amphibole fibers, as they are less easily expelled from the lungs compared to chrysotile fibers (10).

Owing to their differing properties, at least two distinct types of probes, types I and II, are required for the detection of serpentine and amphibole asbestos. The first probe, DksA, was identified as a type I protein with high-affinity binding to chrysotile asbestos derived from bacterial extracts, such as *Escherichia coli* (11). To isolate asbestos-binding proteins, cellular extracts containing various intracellular proteins were mixed with asbestos fibers. During centrifugation, asbestos fibers precipitate along with bound proteins, while unbound proteins are effectively removed through decantation (11). This coprecipitation step allows for the selective isolation of asbestos-binding proteins. The dissociation constant (*K_d_*) of DksA for chrysotile is approximately 3.5 nM, indicating an affinity comparable to that of the antibodies, as determined by Scatchard analysis (11).

These five types of amphibole asbestos share similar crystal structures and surface properties, allowing for the use of amosite in the screening and development of a cross-reactive probe for amphibole asbestos. Screening of the *E. coli* cellular protein library revealed type II proteins (GatZ and H-NS) that bind to all types of amphibole asbestos (12), as well as some non-asbestos fibers, including wollastonite (CaSiO_3_), an increasingly common asbestos substitute. A possible explanation for the lack of protein specificity is the presence of multiple binding domains with affinities for various fiber types. A 31-amino-acid peptide responsible for the affinity of the H-NS protein to amosite was identified by analyzing the binding properties of various deletion domains, which resulted in a more specific binding affinity to amphibole asbestos (13).

### 2.1 Modification of asbestos-binding proteins with fluorescent dyes

Asbestos does not exhibit natural fluorescence under visual light excitation; however, it can be fluorescently stained using fluorescently labeled asbestos-binding proteins, making it visible under FM. These asbestos-binding proteins are produced using standard *E. coli* recombinant techniques (11, 13). To label the recombinant asbestos-binding proteins with Cy3 fluorescent dye, a bright red-orange (~570 nm) fluorescent dye excited by green light (~550 nm) was used following the standard protocol (12). The proteins were mixed with commercially available Cy3-succinimidyl ester dye, which reacts with the amino groups of the proteins. After the reaction, unreacted Cy3 dye was removed to purify the labeled proteins using size-exclusion chromatography. Additionally, Alexa Fluor 488-succinimidyl ester, a green (~520 nm) fluorescent dye excited by blue light (~490 nm), was also used to chemically modify asbestos-binding proteins.

Alternatively, streptavidin, with strong and specific affinity for biotin, was used to create fluorescent probes for asbestos (13). Streptavidin-Cy2, a commercially available conjugate of streptavidin and the green fluorescent dye Cy2, readily binds to biotin-labeled proteins or peptides due to the highly stable, non-covalent streptavidin-biotin interaction. Biotin-labeled asbestos-binding proteins or peptides were produced using *E. coli* recombinant techniques. By mixing streptavidin-Cy2 with a biotin-labeled asbestos-binding proteins or peptides, fluorescent probes for asbestos were successfully generated.

**Figure 2:**
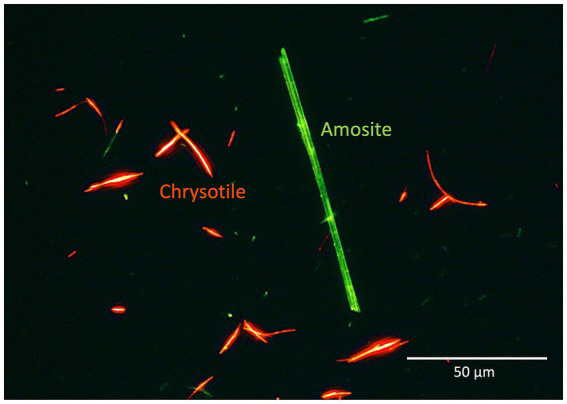
Multicolor and differential imaging of two types of asbestos fibers using both type I and II probes. The type I and II probes were labeled with red and green fluorescence, respectively. The image was processed using the photo editing software “Photos” to enhance the red and green fluorescence based on the original image from Kuroda et al. (15) with permission.

By labeling type I and II proteins with different fluorescent colors, multicolor and differential imaging can be achieved, enabling simultaneous visualization of chrysotile and amphibole fibers in a single field of view (Figure 2) (12). This approach enables clearer discrimination between the two types of asbestos fibers when using a high-performance fluorescence microscope equipped with a double-bandpass filter.

### 2.2 Specificity of fluorescently labeled asbestos-binding proteins

**Figure 3:**
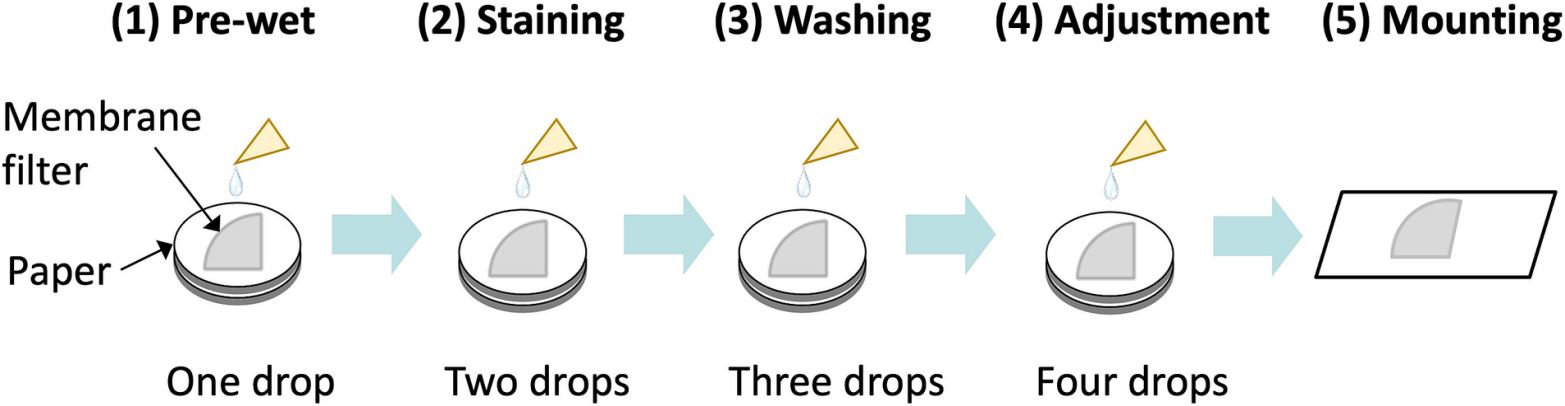
Overview of fluorescent staining of asbestos on a filter membrane. The sample membrane filter wedge (one-fourth of a 25 mm membrane in this illustration) was put on chromatography paper, followed by pre-wet, staining, washing, and adjustment steps prior to mounting of the sample.

To assess the specificity of fluorescently labeled asbestos-binding proteins, binding tests were conducted on chrysotile, all types of amphibole asbestos, and 10 common non-asbestos fibrous materials (Table 1) provided by the Japan Fibrous Material Research Association (14). The 10 non-asbestos materials included nearly all widely used asbestos substitutes in the construction industry, as well as several fiber types from other industries. To stain an asbestos-dispersed membrane filter, the membrane filter was first pre-wetted with Assay Buffer A [0.1 M sodium carbonate buffer (pH 9.5), 1% Tween80] (Figure 3). Subsequently, Assay Buffer A containing 20 nM of fluorescent probes was applied. The membrane filter was then washed with Assay Buffer A to remove the unreacted fluorescent probes. An additional adjustment step was performed to contribute to the washing and the filter transparent processes (see Section 3 for PCM-FM method). During the staining and washing steps, the membrane filter was placed on Whatman Grade No. 3MM chromatography paper to facilitate capillary removal of excess liquid. Finally, the membrane filter was mounted on a glass slide, enclosed in a drop of a 70% (w/w) glycerol solution, and sealed with a coverslip (Figure 3).

**Table 1:** Specificity of asbestos-binding proteins (Type I and II).

Fiber		Type I	Type II
Asbestos	Chrysotile (JAWE, UICC)	Bound	ND
	Crocidolite (JASFM)	ND	Bound
	Amosite (JASFM)	ND	Bound
	Anthophyllite (NIST1867)	ND	Bound
	Tremolite (NIST1867)	ND	Bound
	Actinolite (NIST1867)	ND	Bound
Non-asbestos	Glass wool (JASFM)	ND	ND
	Fine glass fiber (JASFM)	ND	ND
	Rockwool (JASFM)	ND	ND
	Fire proof fiber 1 (JASFM)	ND	ND
	Fire proof fiber 2 (JASFM)	ND	ND
	Aluminum silicate fiber (JASFM)	ND	ND
	Titanium potassium (JASFM)	ND	ND
	Silicon carbide whisker (JASFM)	Bound	Bound
	Titanium oxide whisker (JASFM)	ND	ND
	Wollastonite (JASFM)	ND	ND

Fiber samples were dispersed in deionized water and filtered through a nitrocellulose membrane filter. The filters were then air-dried and stained with the fluorescent reagent as shown in Figure 3. The binding specificity of the fluorescent reagent was evaluated using the PCM-FM method. The fibers were first observed in the PCM mode, and then the mode was switched to FM to assess reagent binding. Fibers visible in both PCM and FM modes were classified as “Bound,” indicating successful binding of the fluorescent reagent. Fibers that appeared only under PCM and not under FM were recorded as “ND” (not detected), suggesting an absence of binding. JAWE, Japan Association for Working Environment Measurement; UICC, Union International Control Cancer; JASFM, Japan Association for the Study of Fiber Materials; NIST, National Institute of Standards and Technology.

**Figure 4:**
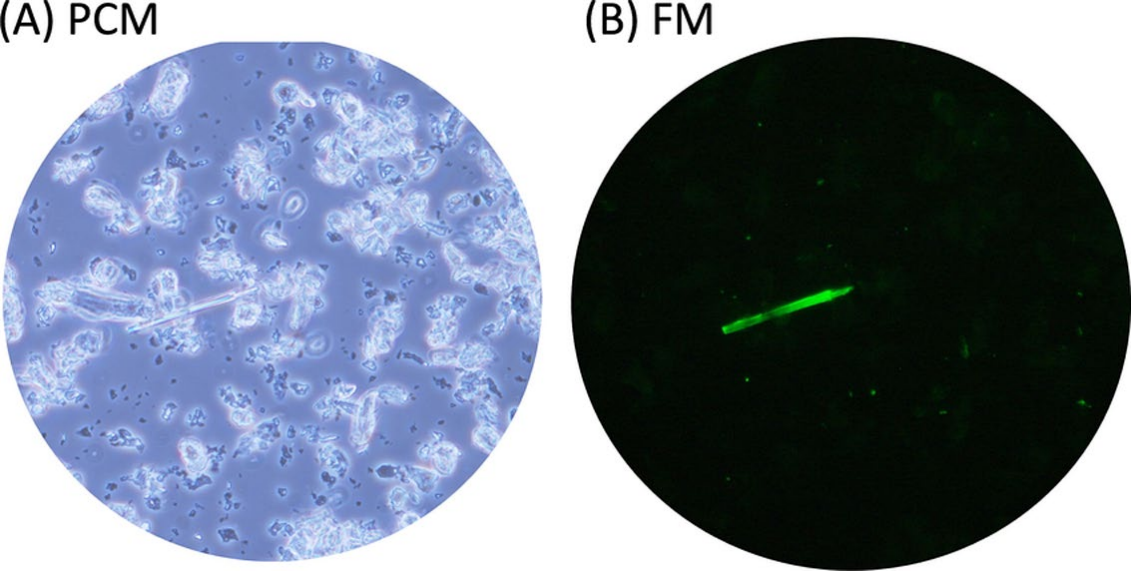
Rapid detection of asbestos contamination in talc. Powdered talc containing asbestos was mixed with the fluorescent-asbestos probes. Then the mixture was applied to a glass slide and covered with a cover slip. The same fields of view were captured under both PCM **(A)** and FM **(B)** modes.

The type I probe selectively bound to chrysotile but not to amphibole asbestos or any non-asbestos fibers, except for silicon carbide whiskers, demonstrating its high specificity for chrysotile asbestos (Table 1). The type I probe can specifically distinguish chrysotile from other serpentine minerals, such as antigorite and lizardite. In contrast, the type II probe exhibited sufficient affinity and specificity for detecting all five types of amphibole asbestos and could distinguish them from 10 common non-asbestos fibrous materials, except for silicon carbide whiskers (13, 15) (Table 1). The asbestos probes did not bind to materials such as talc. Recently, our team demonstrated that the FM method enables rapid detection of asbestos contamination in talc, as only asbestos fibers fluoresce, even in the presence of numerous talc particles (Figure 4) (16).

An alanine scanning experiment on the H-NS peptide (type II), in which individual amino acids in the peptide sequence were replaced with alanine—a small, non-reactive amino acid—revealed that the positively charged side chains of the lysine residues were primarily responsible for the affinity of the probe to amphibole asbestos. This suggests that the binding mechanism likely involves electrostatic interactions between lysine and the negatively charged surface of amphibole asbestos (13). However, since the peptide can distinguish between the negatively charged surfaces of glass wool, Rockwool, and amphibole asbestos, the binding mechanism may also involve other interactions such as hydrophobic and hydrogen bonding (15).

### 2.3 Sensitivity of fluorescently labeled asbestos-binding proteins

To compare the sensitivities of the FM and PCM methods, fiber counting was conducted using the same asbestos-dispersed membrane filter. The FM image was clearer than the PCM image in which some of the fluorescent fibers were invisible. For chrysotile, FM counts were approximately seven times higher than PCM counts (Table 2), likely due to the presence of a significant number of fibers smaller than 0.25 μm, which are undetectable with PCM. In contrast, the FM and PCM counts for amosite and crocidolite were nearly identical, suggesting no apparent increase in sensitivity (Table 2). The limited visibility of thin chrysotile fibers under PCM is likely due to the small refractive index difference between chrysotile (1.53–1.55) and the acetone-treated nitrocellulose filter (approximately 1.43) (17). However, for amosite, the larger refractive index difference (1.64–1.68) results in a greater phase shift, enhancing the visibility of thin amosite fibers under PCM (17).

**Table 2:** Sensitivity of FM method compared to PCM.

Sample	FM		PCM		Ratio (FM/PCM)
	Field of view	Number	Field of view	Number	
Chrysotile Sample 1	17	206	50	73	7.03
	14	201.5	50	112	
Chrysotile Sample 2	15	205	50	94.5	6.92
	19	210.5	50	82	
Amosite Sample 1	40	202	38	202	0.96
	37	205	36	202	
Amosite Sample 2	37	205	35	202	0.99
	29	203	30	203	
Crocidolite Sample 1	30	205.5	29	206	0.99
	29	202	30	204	
Crocidolite Sample 2	29	201.5	30	200.5	1.05
	30	210	31	205	

**Figure 5:**
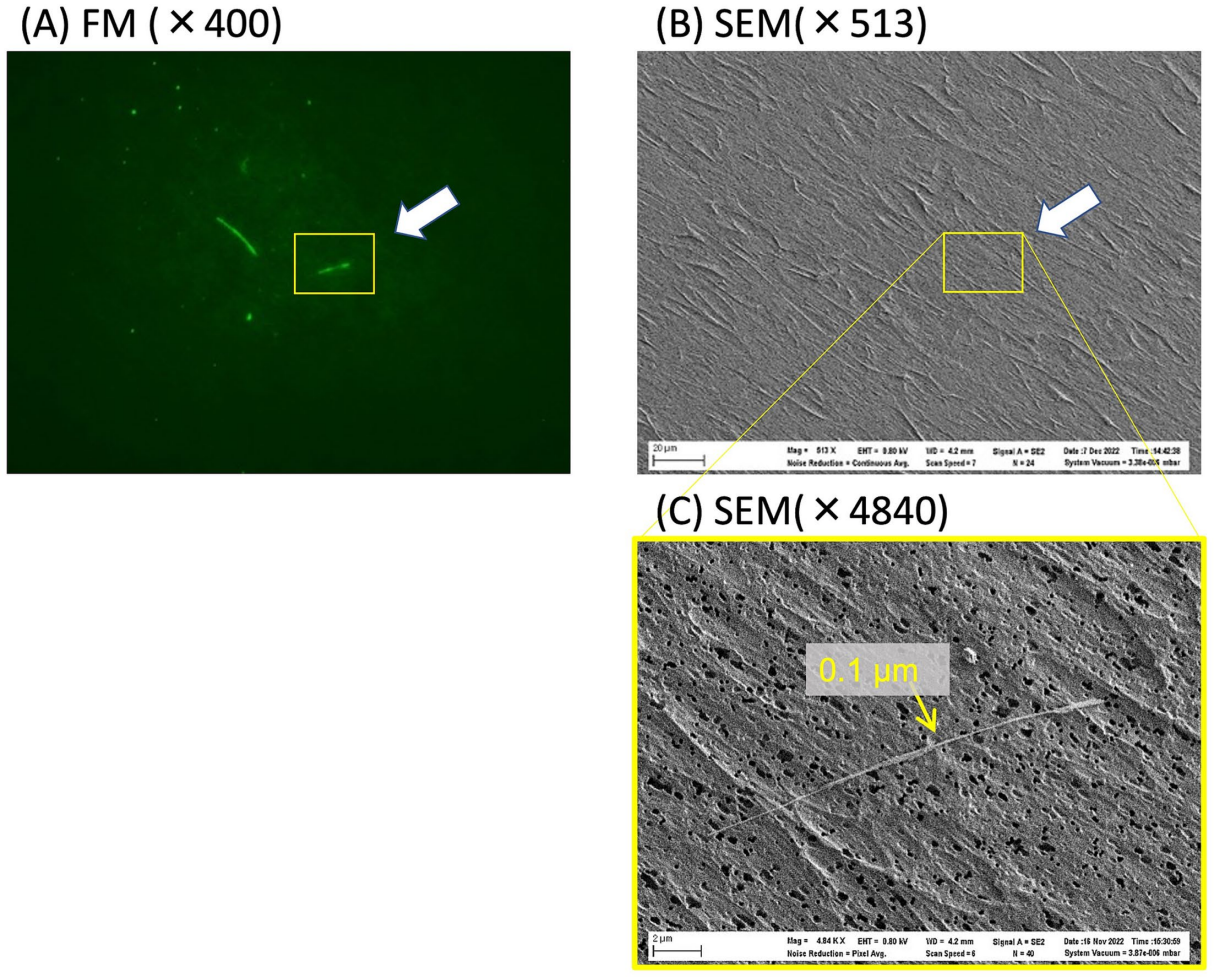
FM and SEM images of asbestos dispersed on a polycarbonate membrane filter. **(A)** The locations of fluorescently stained fibers were recorded under FM and then repositioned and observed again under SEM **(B)**. The yellow rectangle in **(B)** was magnified ×4,840 under SEM **(C)** to determine the diameter of the fluorescent fiber.

Correlative microscopy relies on a specially designed sample holder that is shared between FM and SEM and includes a motorized stage and automated alignment. This technique can be used to examine the same field of view under both FM and SEM platforms and thus directly measure the diameter of the thinnest fibers visible in the FM (18). Although fluorescent fibers were observed under FM (Figure 5A), they were difficult to detect at low magnification (×513) under SEM (Figure 5B). The fine asbestos fiber had a diameter of 0.1 μm under high magnification (×4,840) SEM (Figure 5C). In this case, an almost 10-fold increase in magnification was necessary to detect such thin asbestos fibers under SEM, suggesting that an almost 100-fold increase in view would be required to analyze an area of the same size under SEM, compared with visualization using FM. This FM characteristic reduced the time required to detect fine asbestos contamination.

Currently, the commonly used PCM method cannot reliably detect the most toxic fibers (with diameter <0.25 μm for lung cancer and asbestosis, <0.1 μm for mesothelioma (19); with diameter <0.3 μm for lung cancer and asbestosis, <0.2 μm for mesothelioma (9)). Therefore, either SEM or TEM analysis is necessary to estimate the number of thin fibers. The diameter of the thinnest chrysotile fluorescent fibers, as estimated by SEM, was approximately 30–35 nm, similar to the reported dimensions of single chrysotile fibrils (20). This result demonstrates that the FM sensitivity is sufficient for detecting single chrysotile fibrils. However, the fluorescence intensity can vary depending on the light source, performance of the excitation and emission filters, and camera settings used for image capture. Even a single chrysotile fibril became visible with a stronger light source. Recently, the use of a fluorescence quality control slide was proposed as a standard for normalizing asbestos detection sensitivity (16). This slide includes 16 rectangles (22.5 μm × 1.5 μm) of varying fluorescence intensities, which remain stable over time and unaffected by conventional imaging light sources. Consequently, these slides calibrate the fluorescence intensity levels of various fluorescence microscopes, ensuring consistent detection sensitivity (16).

However, it is important to note that the resolution of FM is limited by optical diffraction, making it difficult to accurately determine the diameters of the nano-scale fibers. Under FM, these nano-scale fibers often appear thicker than those under SEM, which is a limitation of the FM method. For cases requiring precise fiber measurements, TEM or SEM analyses are indispensable. The use of a correlative microscopy system offers a significant advantage, as it enables the same fluorescent fibers to be analyzed under SEM, combining the strengths of both techniques for more accurate characterization (21).

## 3 PCM-FM method

**Figure 6:**
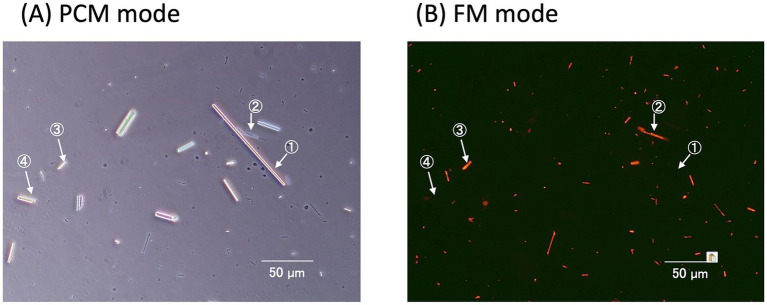
PCM-FM method. For each field of view, PCM is used for the initial observation **(A)**. After noting the number and position of the countable fibers under the fiber counting rules of NIOSH Method 7,400, the analyst switches to FM view to confirm fluorescent staining of the identified fibers **(B)**. The non-fluorescent fibers (numbers 1 and 4) invisible on the FM image would be “non-asbestos” and excluded from the PCM counts.

The counting method for asbestos, commonly used in epidemiological studies, relies on the PCM. As mentioned earlier, the FM method has demonstrated greater sensitivity for detecting chrysotile than the PCM, which may lead to discrepancies in asbestos risk assessment. To address this, a complementary differential counting method has been proposed (21). This method utilizes a microscope that can seamlessly switch between PCM and FM to analyze the same fields of view (Figure 1C). In the combined PCM-FM method, the membrane filter was rendered transparent by exposure to a stream of acetone vapor after the fluorescent staining. First, the PCM mode was used to count the fibers, after which the FM mode was switched to without shifting the field of view to distinguish the asbestos from non-asbestos fibers (Figure 6). For instance, fibers 2 and 3 exhibited fluorescence, while fibers 1 and 4 did not, indicating that fibers 1 and 4 are non-asbestos. Although the standalone FM method is more sensitive than PCM for chrysotile fiber counting, the PCM-FM differential counting approach complements the PCM analysis and is fully compatible with PCM-based epidemiological data.

**Figure 7:**
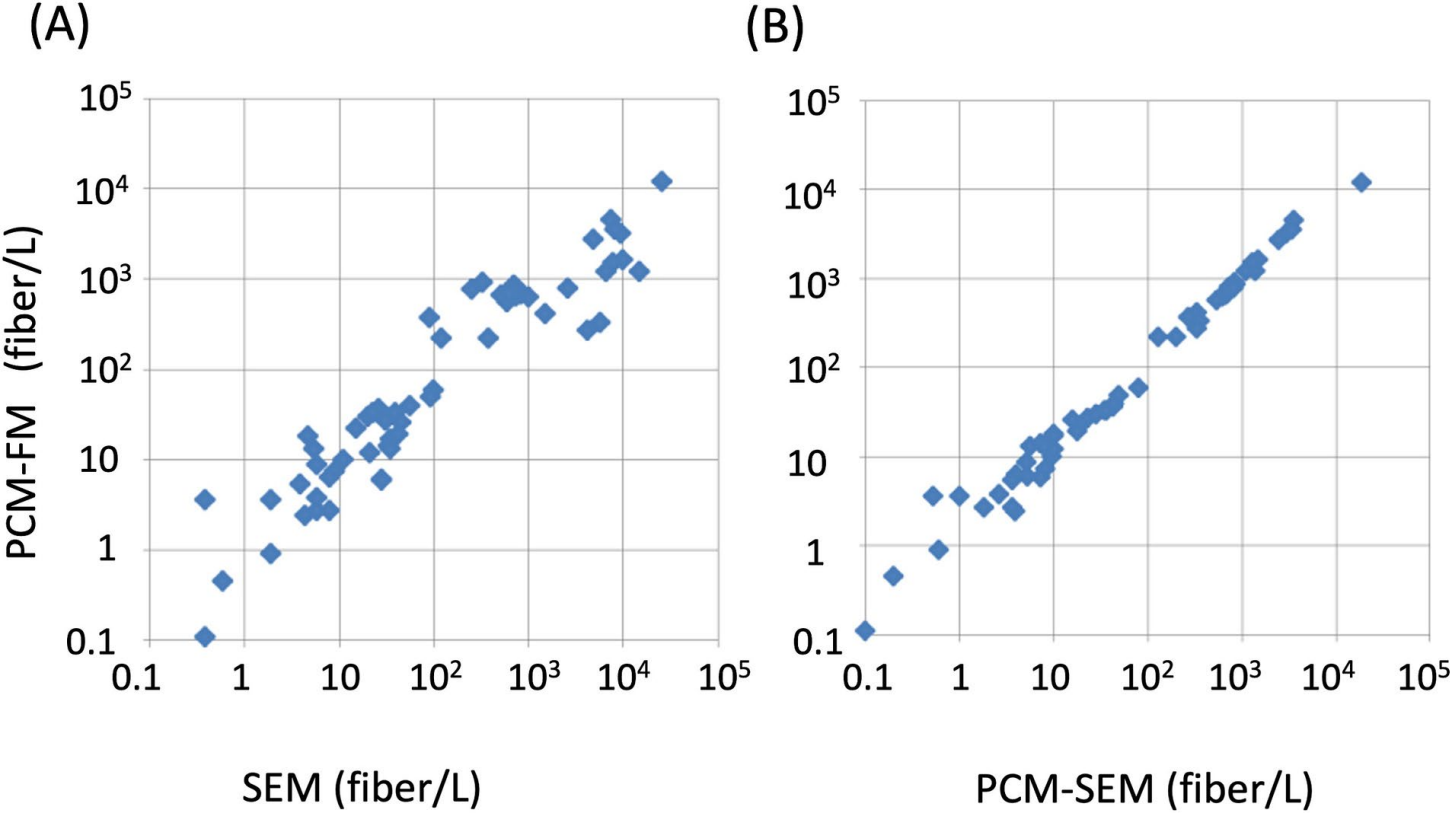
Correlation between PCM-FM and SEM-based methods. The fiber concentration determined using the PCM-FM method was plotted over these using the SEM method **(A)** and PCM counts corrected by SEM asbestos fraction (PCM-SEM) **(B)**. Membrane filter samples containing chrysotile and amphibole asbestos were collected from demolition sites across Japan and prepared from asbestos-containing materials. This figure was reproduced from Nishimura et al. (21) with permission.

In Japan, differential counting by electron microscopy (either SEM or TEM, equipped with EDX or SAED) is mandatory for samples with PCM fiber counts exceeding 1 f/L, after which PCM counts are disregarded in favor of electron microscopy results (22). The second method, relying on electron microscopy, determines the fraction (ratio) of asbestos fibers, which complements the PCM counts. This approach is used in the US, where TEM analysis complements the PCM counts (23). The PCM-FM method was compared with the SEM-based method using airborne dust samples from demolition sites in Japan. The PCM-FM counts correlated highly with the PCM counts adjusted by applying the SEM asbestos fraction (Figure 7).

## 4 On-site detection of airborne asbestos

**Figure 8:**
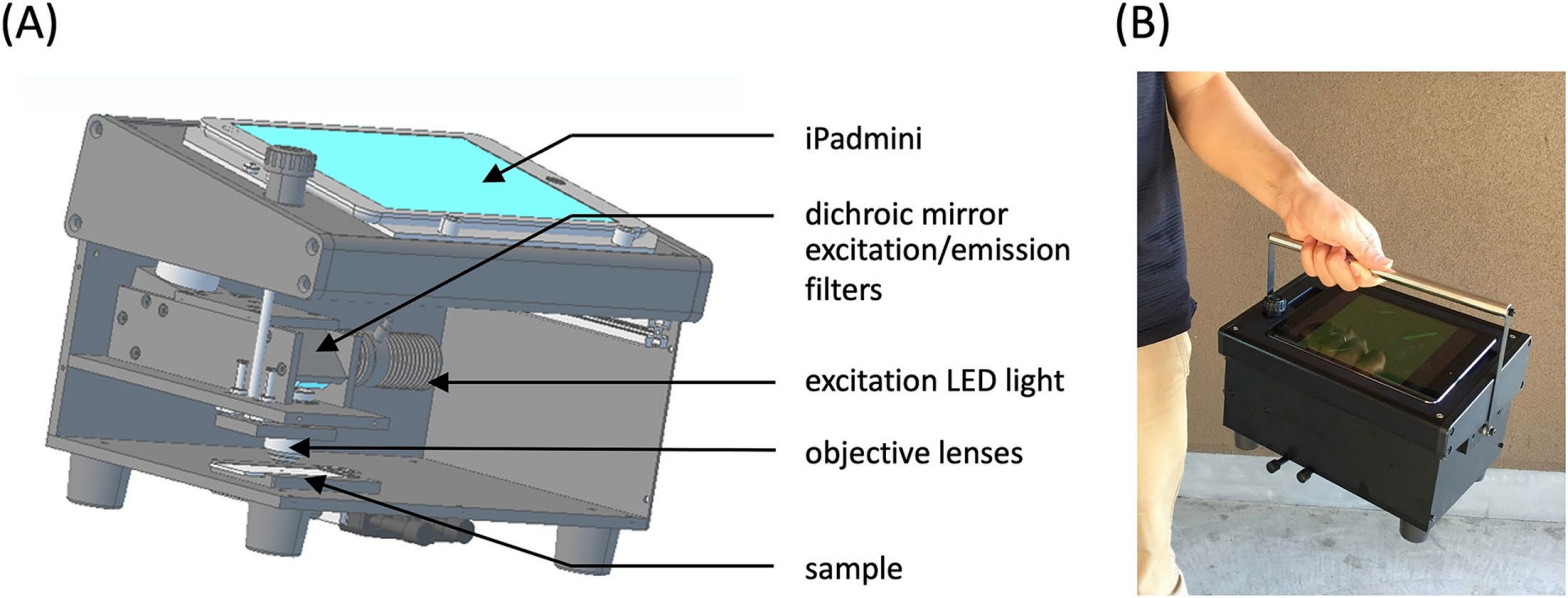
Portable fluorescence microscope. **(A)** Illustration of the inner structure of the portable fluorescence microscope. **(B)** Photograph of the portable fluorescence microscope. This photograph was reproduced from Kuroda et al. (15) with permission.

Although the FM method enables more sensitive and selective detection of asbestos than the conventional PCM method, laboratory-grade fluorescence microscopes are unsuitable for harsh environments such as demolition sites. This necessitates a robust, portable, and battery-operated microscope for on-site asbestos detection. Smartphones and tablets, with their advanced optics, computational power, data connectivity, and low cost, provide an ideal platform for microscopy (24–26). A portable fluorescence microscope was developed that integrates objective lenses, excitation LED light, excitation/emission filters, and a dichroic mirror, all housed within a hard aluminum alloy box to ensure usability under harsh and bright conditions (Figure 8A). The optical components were aligned with the back camera of an iPad mini, which served as the detachable device for monitoring and saving microscopic fluorescence images. Sample images can be accessed remotely from an asbestos laboratory in real-time via iPad connectivity. A handle was added to the portable fluorescence microscope to enhance the portability, bringing the total weight to approximately 3.8 Kg (Figure 8B).

Fluorescent fibers longer than 5 μm and thinner than 3 μm with aspect ratios larger than 3:1 were counted as asbestos fibers under the portable FM. If necessary, the field of view (390 μm × 390 μm) could be enlarged five-fold (to 78 μm × 78 μm) on the iPad display. The total number of fluorescent fibers over the area of the membrane filter (13 mm in diameter, 516 views) was counted. The fiber concentration was calculated as the total number of fibers divided by the amount of air collected. Our team measured airborne asbestos concentrations using the portable fluorescence microscope at 20 demolition sites across Japan. Typically, asbestos detection can be completed within an hour, with approximately 20 min for sampling and staining and 40 min for counting of the fibers. While most demolition sites showed fiber concentrations below 1 fiber/L, one site had asbestos fibers (5.2 fibers/L) leaked from the containment area into an adjacent chamber. The filter from this sample was taken back to the laboratory, where SEM–EDX analysis confirmed that the fibers were amosite. The portable FM method has proven to be a promising rapid screening tool for detecting high concentrations of airborne asbestos leakage in asbestos work zones. Additional details on the instrumentation and methodology of the portable FM, as well as validation data and comparisons with the SEM method can be found in Supplementary Figure S1. In 2022, the revised Asbestos Monitoring Manual of Japan included the portable FM method as a screening tool for asbestos leakage at demolition sites (22), reflecting a policy that prioritizes simple, rapid, onsite asbestos detection methods to mitigate the risk of asbestos dispersion, even with slightly reduced accuracy.

## 5 Live-cell imaging of asbestos

**Figure 9:**
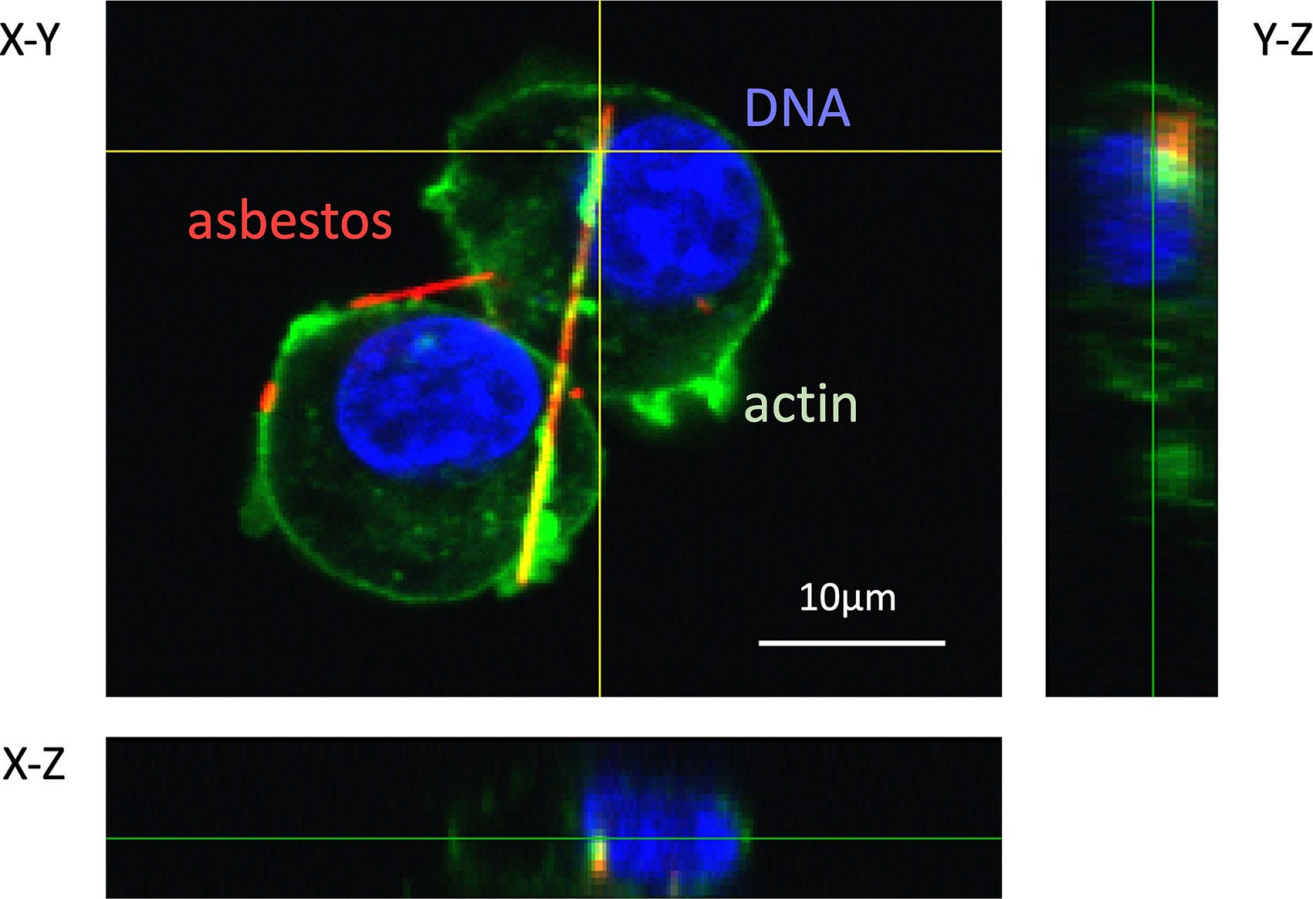
Confocal laser scanning fluorescence microscopy image of phagocytosis of fluorescently labeled asbestos fibers by RAW 264.7 cells. The complex of streptavidin-Cy3 (red) and asbestos-binding protein (type II) was used to prepare fluorescently labeled amosite. The actin cytoskeleton and nuclei of the cells were stained with ActinGreen™ 488 (green) and DAPI (blue), respectively. Z-axis images at vertical and horizontal yellow lines were extracted from 3D images, and indicate right and bottom positions, respectively. This image was reproduced from Ishida et al. (29), which is an open access article.

Most cases of mesothelioma result from exposure to asbestos fibers in the environment or occupational ambient air. However, the following questions regarding asbestos toxicity remain partially unanswered: (i) why asbestos entering the alveoli during respiration exerts toxicity in the pleura; and (ii) how asbestos causes mesothelioma, even though human mesothelial cells are easily killed upon exposure to asbestos. For the latter, current evidence suggests that frustrated phagocytosis of asbestos fibers by macrophages prolongs inflammation and creates a “mutagenic microenvironment” that promotes the malignant transformation of mesothelial cells. Epidemiological and genetic studies have proposed a carcinogenic model in which mutations in BRCA1-associated protein 1 suppress cell death in mesothelial cells and increase genomic instability in the mutagenic microenvironment. This leads to additional mutations in genes such as *CDKN2A* [p16], *NF2, TP53*, *LATS2*, and *SETD2*—hallmarks of mesothelioma (27, 28). Therefore, one of the earliest and most critical events in asbestos-induced toxicity is the physical interaction between asbestos fibers and living cells. This interaction includes fiber adhesion to the cell surface, internalization, intracellular trafficking, and the resulting cellular response. When fluorescently labeled asbestos fibers were used in conjunction with fluorescent markers for cellular structures (e.g., plasma membrane, actin cytoskeleton, endosomes, and lysosomes), FM enabled visualization of these processes. As shown in Figure 9, internalized asbestos fibers co-localized with actin (green), forming an orange signal due to the overlap of red (Cy3-labeled fibers) and green (actin) fluorescence, indicating their enclosure within actin-rich phagosomes (29). In contrast, fibers merely interacting with the cell membrane retained their red appearance, suggesting that they had not been internalized. These findings highlight the ability of FM to distinguish between ingested fibers and those only attached to the cell surface, demonstrating the method’s utility in visualizing actin-mediated phagocytosis of asbestos.

Regarding the former question about asbestos entering the alveoli during respiration and exerting toxicity in the pleura, the mechanism of transfer of inhaled asbestos from the alveoli to the pleura remains obscure. Fluorescent labeling of asbestos fibers enables researchers to monitor the localization, uptake, retention, and transfer of fibers over time. Live-cell imaging of asbestos has shown that macrophages carrying internalized asbestos remain motile (30), indicating their potential role in transporting asbestos from the alveoli to the pleura. Overall, FM provides vital insights into how asbestos fibers interact with cells at structural and functional levels, helping to elucidate the mechanisms underlying fiber toxicity, inflammation, transfer, and eventual disease development.

## 6 Conclusion and future tasks

The application of FM for detecting micro- and nano-scale inorganic materials has traditionally been limited owing to the lack of specific fluorescent probes. However, research by our team and others (31, 32) has demonstrated that asbestos-binding proteins effectively serve as fluorescent probes, enhancing the sensitivity and selectivity of FM for detecting asbestos fibers in air, liquids, and materials. Notably, FM can identify nano-scale fibers at lower magnifications, reducing the labor costs for asbestos contamination detection. Considering these advantages, two FM-based methods have been developed: (i) the combined PCM-FM method, a differential counting approach that complements PCM analysis and remains fully compatible with PCM-based epidemiological data, and (ii) the portable FM method, which shows significant potential for rapid on-site asbestos screening at demolition sites. Furthermore, FM may facilitate the live-cell imaging of asbestos interactions, broadening its applications in asbestos research.

Despite promising results, some challenges persist. Asbestos cannot be definitively identified based solely on fluorescent probes, and detailed morphological analyses are necessary to identify asbestos. Furthermore, additional research is needed to assess the cross-reactivity of these probes. In a previous study, the author’s research team found that approximately 95% of fluorescently stained fibers in demolition site samples were correctly identified as asbestos (21). However, some false-positive fibers were observed, including surface-coated mineral wool fibers. Porous or permeable surface coatings can lead to nonspecific probe adsorption, resulting in nonspecific staining. The second largest group of fluorescently stained non-asbestos fibers is comprised of organic microfibers, which may inherently contain or be coated with natural or artificial fluorescent dyes, causing autofluorescence even without additional fluorescent staining (21). Autofluorescence typically exhibits a broad range of excitation and emission wavelengths, whereas the fluorescent dyes used for asbestos probes are activated only at specific excitation wavelengths. Consequently, briefly switching to an excitation wavelength that does not activate the asbestos probes enables analysts to identify the fibers that remain illuminated as autofluorescent non-asbestos fibers.

Another significant challenge lies in understanding the precise binding mechanisms of asbestos-binding proteins. The mechanisms by which these proteins selectively recognize chrysotile among serpentine minerals, amphiboles, and man-made fibers remain unclear. Resolving this issue could further improve the reliability of the FM method. Currently, fluorescent probes cannot distinguish amphibole asbestos from cleavage fragments, which are considered less toxic (8). However, as mentioned in the introduction, peptides capable of differentiating GaAs crystal faces have been reported (7). The development of more specific peptide probes in the future may allow for the distinction of amphibole asbestos from cleavage fragments, further improving reliability.

Our team is in the process of developing a fully automated process for airborne asbestos detection that integrates air sampling, staining, and analysis into a seamless workflow. Unlike traditional PCM methods, the standalone FM method does not require a transparent filter, allowing for continuous and efficient airborne asbestos monitoring. This system uses a long-strip membrane filter for air sampling, followed by automatic staining, transfer to an FM setup, and image capture. The fluorescent fibers are then identified and counted using specialized software (33, 34), creating a streamlined and highly efficient automated process for detecting airborne asbestos. This project is currently supported by the Japan Environmental Ministry.
