# Supplementary material for: Fluorescent labeling of asbestos fiber for enhanced asbestos detection under fluorescence microscopy
Source: Front Public Health. 2025 May 21;13:1568581. doi: 10.3389/fpubh.2025.1568581 (PMC12133516; doi:10.3389/fpubh.2025.1568581)
Supplement: Supplementary file 1 [file Data_Sheet_1.PDF]

Fluorescent Labeling of Asbestos Fiber for Enhanced Asbestos Detection under Fluorescence Microscopy

Akio Kuroda
Graduate School of Integrated Sciences for Life, Hiroshima University, Higashi-Hiroshima, Hiroshima 739-8530, Japan

* Correspondence:
Akio Kuroda
akuroda@hiroshima-u.ac.jp

Supplementary Material

1. Details on the instrumentation and methodology of the portable FM

Instrumentation: The portable FM integrates an optical system (objective lens: 40×/0.65 NA; LED: 470 nm; excitation filter: 480 nm; dichroic mirror: 505 nm; emission filter: 515 nm long-pass) within a compact, box-shaped enclosure equipped with a carrying handle and powered by a mobile battery. The optical pathway is focused to a built-in rear camera of an iPad mini 5 (Apple Inc.) on the top panel, and fluorescent images can be saved in the iPad's memory.

Methodology: Fluorescent staining for FM analysis was conducted as described in the main text (lines 129–137) and illustrated in Figure 3, using the Asbester Air2 kit provided by Siliconbio Inc. (Hiroshima, Japan).

Validation: The accuracy of asbestos detection was compared between the portable FM and SEM. Artificial membrane filters containing chrysotile, amosite, or crocidolite were prepared from finely crushed asbestos-containing materials. After fluorescent staining, each filter was divided and analyzed using both the portable FM and SEM. Under the portable FM, fluorescent fibers longer than 5 µm, thinner than 3 µm, and with aspect ratios greater than 3:1 were counted. In the SEM analysis, fibers were counted at ×1,000 magnification and identified using energy-dispersive X-ray spectroscopy. A strong correlation was observed between the number of fluorescent fibers (FM) and asbestos fibers (SEM) per 1 mm^2 ($r = 0.94$; Figure S1A). The mean relative difference in asbestos concentrations between the two methods was +6.9%, with a 1.96 SD range of −52.3% to +66.0% (Figure S1B).

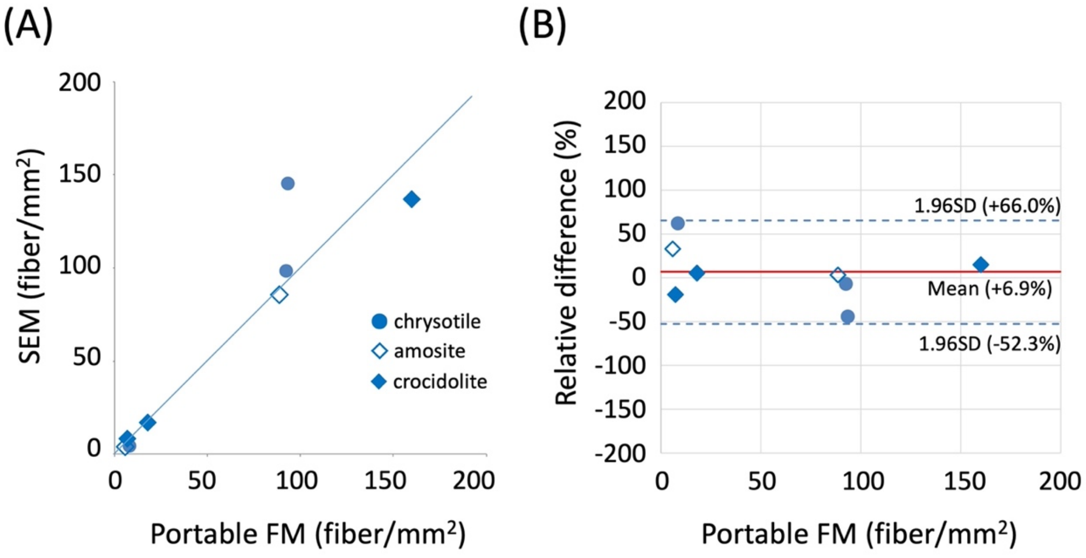

Figure S1: Correlation (A) and relative difference (B) between portable FM and SEM methods. The relative difference was calculated as: (FM fiber/mm² – SEM fiber/mm²) / mean of FM and SEM fiber/mm².

Supplementary reference:

Kuroda A, Nishimura T, Ishida T. Validation of asbestos analysis using a portable fluorescence microscopy [in Japanese]. Fibrous material research. (2020) 7: 56-60.
